# Editorial: Corneal transplantation and eye banking

**DOI:** 10.3389/fmed.2022.983580

**Published:** 2022-07-21

**Authors:** Vito Romano, Stefano Ferrari, Hannah J. Levis, Mohit Parekh

**Affiliations:** ^1^Department of Medical and Surgical Specialties, Radiological Specialties and Public Health, University of Brescia, Brescia, Italy; ^2^International Center for Ocular Physiopathology, Fondazione Banca degli Occhi del Veneto Onlus, Venice, Italy; ^3^Institute of Life Course and Medical Sciences, University of Liverpool, Liverpool, United Kingdom; ^4^Institute of Ophthalmology, University College London, London, United Kingdom

**Keywords:** corneal transplant, cornea donor, eye bank, lamellar keratoplasty, cornea

Cornea, the front transparent layer of the eye, is responsible for vision clarity. Disease or dysfunction in any layer of this multilayer tissue can lead to corneal blindness in addition to pain and discomfort. Corneal transplantation is the most popular choice of treatment where a healthy donor graft obtained from a cadaver is harvested, stored or processed in an eye bank and used to replace the diseased host tissue (1). However, human cadaveric corneal tissues have a worldwide shortage, so researchers are finding alternative solutions to treat corneal disorders (2). Improved surgical techniques, graft restoration procedures, cell and molecular based treatment options, and tissue alternatives have all contributed to the advancement in the field of corneal transplant and eye banking (3). In addition, since the pandemic impacted tissue procurement significantly, a huge waiting list was observed due to lack of tissues for elective surgeries (4, 5). However, with significant amount of work to improve the donation rate, the corneal transplantation has resumed and now fully functional with tissue donations being actively pursued.

The studies in this special issue on *Corneal transplantation and eye banking* highlighted recent advances. Novel and long-term clinical outcomes suggested that Boston type 1 KPro can be used for patients with aniridia associated keratopathy (AAK), however, it was suggested that glaucoma and restroprosthetic membrane formation must be considered before transplanting such device in these patients (Dyer et al.). Deep anterior lamellar keratoplasty (DALK) is routinely performed to replace the anterior cornea. Usually, the donor tissue is cut to a desired thickness and the diseased anterior stroma replaced. However, a recent long-term study evaluated a new polymethymethacrylate (PMMA) ring (Neoring) and showed that this synthetic device can be used as a viable, effective, and safe option for pre-Descemet DALK to optimize the post-operative results for moderate-severe keratoconus (Alfonso-Bartolozzi et al.). Gender mismatch in corneal transplantation (6, 7) has been one of the concerns that has not been widely studied. However, a study published in this special issue showed no significant influence of donor-recipient sex- or age-match on graft rejection and failure in eyes that had undergone DALK surgeries (Ong et al.). For endokeratoplasty, pseudophakic status and/or presence of preoperative endothelial folds have been indicated as significant donor risk factors for endothelial failure in non-FECD patients following DSAEK (Nishisako et al.). In addition, many studies have investigated the impact of COVID-19 pandemic on corneal transplantation. Techniques for optimal utilization (8, 9) and extended storage of human donor corneas became crucial during COVID. Therefore, new techniques like femtosecond laser (FSL) incision of rehydrated human donor corneas after air-drying has been evaluated for new and optimized use of donor tissues (Pedrotti et al.). In addition, an Italian study reported that vigorous work and continuous effort toward resuming keratoplasties to a near-normal standard despite the pandemic led to an increase in endokeratoplasties, thus suggesting that the corneal transplantation field is evolving rapidly (Mencucci et al.).

Corneal endothelium has been studied and reviewed extensively. However, due to lack of a standard cell/molecular based treatment approach, many new developments have been observed. Corneal endothelium is the inner monolayer of cells that is considered to be non-proliferative. Hence, it must be maintained as its loss due to disease or dysfunction could lead to corneal blindness. Apart from corneal transplantation, intracameral injection of cultured corneal endothelial cells (10), biomedical engineering of endothelial grafts, novel scaffolds (11) and many other options have been evaluated. An extensive review on diverse array of genes targeted to induce proliferation of corneal endothelial cells has also been compiled in this special issue (Arras et al.). In addition, extracellular vesicles (Parekh et al.) from corneal endothelial cells have shown to inhibit the proliferation of endothelial cells and the miRNAs present in the extracellular vesicles, possibly the exosomes, must be evaluated to target the induction of proliferation by downregulating the causative gene. These studies may help understanding the pathology of corneal endothelial dysfunction and provide further insights in the development of future therapeutic treatment options.

In conclusion, genes modulating the proliferative capacity of endothelial cells, artificial and bio-mimetic corneas, extracellular vesicles, synthetic keratoprosthesis (Holland et al.) with or without the inclusion of biomolecules, advanced bioengineering, 3D corneal bioprinting, biomaterials, artificial corneas, etc. have shown to be promising in advancing the field of *Corneal transplantation and eye banking*.

## Author contributions

VR, SF, HL, and MP: compiled, drafted, reviewed, and approved. All authors contributed to the article and approved the submitted version.

## Conflict of interest

The authors declare that the research was conducted in the absence of any commercial or financial relationships that could be construed as a potential conflict of interest.

## Publisher's note

All claims expressed in this article are solely those of the authors and do not necessarily represent those of their affiliated organizations, or those of the publisher, the editors and the reviewers. Any product that may be evaluated in this article, or claim that may be made by its manufacturer, is not guaranteed or endorsed by the publisher.
